# Recruitment of ipsilateral and contralateral upper limb muscles following stimulation of the cortical motor areas in the monkey

**DOI:** 10.1007/s00221-013-3639-5

**Published:** 2013-07-14

**Authors:** Lynnette R. Montgomery, Wendy J. Herbert, John A. Buford

**Affiliations:** 1 Neuroscience Graduate Studies Program, The Ohio State University, Columbus, OH 43210 USA; 2 Division of Physical Therapy, School of Health and Rehabilitation Sciences, 453 W 10th Ave., 516 Atwell Hall, Columbus, OH 43210 USA

**Keywords:** Reaching, Motor control, Corticospinal, Stimulus trains

## Abstract

It is well established that cortical motor stimulation results in contralateral upper limb (UL) activity. Motor responses are also elicited in the ipsilateral UL, though controversy surrounds the significance of these effects. Evidence suggests that ipsilateral muscle activity is more common following the stimulation of the supplementary motor area (SMA) and dorsal premotor area (PMd), compared to the primary motor cortex (M1), but none of these studies compared effects from all three areas in the same subjects. This has limited our understanding of how these three cortical motor areas influence ipsilateral UL muscle activity. The purpose of this study was to determine the contribution of each of three cortical areas to the production of ipsilateral and contralateral UL. To maximize sensitivity and allow comparison of the effects across cortical areas, we applied the same stimulation parameters (36 pulse stimulus train at 330 Hz) to M1, SMA, and PMd in three adult *M. fascicularis* and recorded electromyographic (EMG) activity from muscles in the trunk and both ULs. Of all muscle responses identified, 24 % were ipsilateral to the stimulation, mostly in proximal muscles. The highest percentage of ipsilateral responses occurred following SMA stimulation. We also observed that PMd stimulation elicited more suppression responses compared with stimulation of M1 and SMA. The results indicate that ipsilateral motor areas provide a significant contribution to cortical activation of the trunk and proximal UL muscles. These understudied pathways may represent a functional substrate for future strategies to shape UL recovery following injury or stroke.

## Introduction

In primates, projections from the three largest motor areas in the frontal cortex, primary motor cortex (M1), supplementary motor area (SMA), and dorsal premotor area (PMd) make up a significant proportion (~70 %) of corticospinal tract fibers (Brinkman and Kuypers 1973; He et al. 1995; Kuypers 1960; Lawrence and Kuypers 1968). Electrical stimulation of these cortical motor areas produces predominantly contralateral movements (Asanuma 1973; Asanuma and Rosen 1972; Boudrias et al. 2010b; Kwan et al. 1978; Penfield 1954; Penfield and Boldrey 1937; Welker et al. 1957; Woolsey et al. 1952). Although the majority of corticospinal fibers originating from the motor cortex terminate in the contralateral spinal cord, about 5–10 % of all corticospinal fibers terminate ipsilaterally (Brosamle and Schwab 1997). These ipsilateral projections are particularly prevalent from SMA, where 23 % of the corticospinal projections are ipsilateral, whereas only 10–13 % of corticospinal projections from M1 are ipsilateral (Dum and Strick 1996; Lacroix et al. 2004; Rosenzweig et al. 2009; Yoshino-Saito et al. 2010). Studies examining muscle activity in both upper limbs (ULs) following SMA stimulation have demonstrated both ipsilateral and bilateral UL movements in addition to purely contralateral movement (Brinkman and Porter 1979; Mitz and Wise 1987; Tanji et al. 1988). Recording studies also report that neural activity in the premotor areas is more likely to be associated with ipsilateral or bilateral UL movement compared to activity from M1 (Kermadi et al. 1998; Tanji et al. 1988).

Many functional UL tasks engage a variety of proximal and distal muscles in both ULs in bilateral movement patterns. Yet, most of the literature on control of movement from cortical motor areas has focused on how activity from one or two of the main cortical motor areas influences isolated distal movements in the contralateral UL (Asanuma and Rosen 1972; Hendrix et al. 2009; Maier et al. 2002; Rizzolatti et al. 1988; Sato and Tanji 1989). In those studies that have recorded electromyographic (EMG) responses in muscles following stimulation of the ipsilateral corticospinal tract or motor cortex, only muscle activity in the distal UL has been recorded (Aizawa et al. 1990; Soteropoulos et al. 2011). No study to date has specifically compared the motor outputs of these three cortical motor areas for the recruitment of muscles, including proximal and distal muscles, in both ULs. This knowledge is important not only because such movements are common during everyday tasks, but also because these ipsilateral and bilateral outputs may have important implications for mechanisms of recovery from stroke. Further, motor outputs from SMA and PMd appear to have a relatively strong influence on proximal UL musculature (Kurata and Tanji 1986; Macpherson et al. 1982; Tanji and Kurata 1979). Thus, a more complete understanding of the functions of these motor areas requires inclusion of proximal UL muscles in the analysis.

The purpose of the present study was to determine the relative contributions of three cortical motor areas, M1, SMA, and PMd, to recruitment of muscles throughout both ULs, including muscles acting from the shoulder girdle to the wrist. Our hypothesis was that sampling responses in this set of muscles, with responses measured by EMG, would reveal stronger contributions to ipsilateral control than previously recognized from the cortical motor areas. Portions of these results have been reported previously (Montgomery et al. 2010).

## Materials and methods

### Subjects

Three male monkeys (*M. fascicularis*) were subjects for this study. All subject care complied with the NIH Guide for the Care and Use of Laboratory Animals, and all protocols were approved by the Institutional Animal Care and Use Committee at The Ohio State University. Subjects performed an instructed-delay, bilateral reaching task as detailed previously (Davidson and Buford 2006), designed to provide detectible changes in muscle activation from baseline EMG. Briefly, the task involved the subject reaching with one hand or the other for a target on a computer screen located in front of him once a cue was given. Stimulation was given at any time during the task as long as the subject was in the process of moving his UL.

Surgeries to implant chronic EMG and recording chambers were performed while subjects were under isoflurane anesthesia (1–2 %). Vital signs were monitored throughout the procedure. A craniotomy was made over the left cortical motor areas centered near the precentral dimple (around AP 15 ML-12). A 37 × 37 mm^2^ plastic recording chamber (Alpha Omega, Alpharetta, GA) was placed at a 20° lateral tilt over the craniotomy. Another craniotomy was made over the right occipito-parietal region and a recording chamber was placed over this allowing for access to the pontomedullary reticular formation, for data collection not related to this study. Cranial implants and EMG connectors were embedded in dental acrylic. Chronic EMG implants made from pairs of Teflon-coated stainless steel wires (38 gauge, CoonerWire, Chatsworth, CA) were implanted into 24 selected muscles (listed below). Following surgery, subjects were given analgesics (buprenorphine and ibuprofen) and antibiotics (chlorofenicol or baytril) for several days to prevent postoperative pain and infection.

### Stimulation techniques

Two to four glass coated tungsten microelectrodes (Alpha Omega, Alpharetta, GA) were simultaneously positioned in the cortex, with electrode sites concentrated in the shoulder/elbow regions for each cortical motor area. Once background neural activity was detected, this was taken as a relative reference point for the penetration depth. Cells not related to UL movements were abandoned and other cells sought. Stimulation was applied to sites where neurons with UL-related activity during task performance were found.

Stimulation threshold for evoking movement was determined in response to a 36 pulse stimulus train (biphasic, 200 us per phase, 333 Hz). The objective was to enable direct comparison of responses from the three motor areas with an identical stimulation paradigm. This pulse train duration was chosen for effective stimulation in premotor areas (Mitz and Wise 1987).

Stimulation thresholds were defined as the lowest current (±5 μA) that produced a small but visible muscle twitch. The subject was performing the task as thresholds were determined. Stimulation was delivered using a Master-8 stimulator (A.M.P.I, Jerusalem, Israel) and a current-controlled stimulus isolator (AM-Systems model 2200, Carlsborg, WA). Thresholds varied between cortical motor areas with most currents used in M1 being between 20 and 40 μA, those in SMA being between 50 and 120 μA, and those in PMd being between 30 and 80 μA; these current levels are consistent with previous reports (Hummelsheim et al. 1986; Weinrich and Wise 1982). After the stimulation threshold for each electrode was found, a series of 10–12 stimulus trains was delivered and EMG responses were recorded.

### EMG

Electromyographic (EMG) was recorded using a Power 1401 CED data acquisition system (CED, Cambridge, UK). EMGs were obtained and analyzed from flexor carpi ulnaris (FCU), extensor carpi radialis (ECR), biceps brachii (Bic), triceps brachii (Tri), middle deltoid (MDelt), supraspinatus (supra), upper trapezius (UpTrap), cervical paraspinals (CervPara), pectoralis major (Pec), and latissimus dorsi (Lats). Subjects H and O had EMG leads in sternocleidomastoid (SCM), and subjects N and O had EMG leads in lumbar paraspinals (LumPara). In subject N, EMG from left and right teres major was recorded, and in subject H, EMG from left and right brachioradialis was recorded. However, because data were only collected from one animal for each of these muscles, data from these muscles were eliminated from the analysis.

### Tests for EMG integrity

Every 2 weeks, the EMG leads were tested to confirm whether they were still in the correct muscles. If the threshold current was > 2,000 μA or the muscle response was not in the muscle initially implanted, then the data were omitted from the analysis for time points following this observation. In O, left Lats and left CervPara were removed from the analysis; in H, right CervPara was removed from the analysis. Because the removal of CervPara from these subjects led to an imbalance in the chances for left and right CervPara to produce effects, all data for CervPara for both subjects were eliminated from the analysis. EMG implants in H for left FCU and ECR failed near the end of the study, so these data were removed from the analysis for the last three sessions. Left and right LumPara were removed from data analysis for N for the last two sessions.

To test for EMG cross talk, we used an approach described by Cheney and Fetz (1985). A customized script was written using Spike 2 software (CED, Cambridge, UK). Representative files were chosen from the first and last few weeks of the experiment. EMG waveforms resembling single motor unit action potentials were located for each muscle, and these were then used as triggers for the averaging of activity in all muscles studied. If the waveform from the muscle used as the trigger was evident in another muscle, and that waveform had amplitude greater than 15 % of that in the triggering muscle, this was considered cross talk. There was only one case that met this test; in subject H, right MDelt was removed from the analysis due to cross talk with right supra.

### Data analysis

Each EMG waveform was a rectified average of the evoked activity that occurred during the 10–12 stimulus trains applied through each cortical electrode over a 300 ms window. An 89-ms period, before stimulus onset, was used to calculate the EMG baseline mean and standard deviation (s.d.) for all muscles, for each file. The baseline period ended 10 ms before stimulus onset, to avoid any effect of stimulus artifact. Facilitation was detected by a conventional approach, defining facilitation as the period where the EMG level was elevated by at least 2 s.d. above baseline and where the increase reached a peak of at least 4 s.d. above the mean.

Suppression was more difficult to detect because, in many cases, there was relatively little muscle activity at the time of stimulation. In examining the EMG records visually, we found many instances that appeared to be a clear case of suppression, but which the conventional 2 s.d. criteria could not detect. In these cases, we observed that although the mean EMG level decreased only slightly, the variability of the EMG level was markedly reduced. In order to objectively measure this so as to detect these instances of apparent suppression, we developed a new approach.

Electromyographic (EMG) data were processed to replot the data as the s.d. of the EMG level taken over a moving 5 ms window. This allowed us to measure variations in EMG level, which added an additional parameter for the detection of suppression. Two of the authors (LRM, JAB) performed sensitivity and specificity analyses of all suppression responses to compare the visual inspection with a variety of objective criteria. We repeated the sensitivity and specificity analyses for all facilitation responses to compare the conventional objective criteria to our visual inspection.

**Fig. 1:**
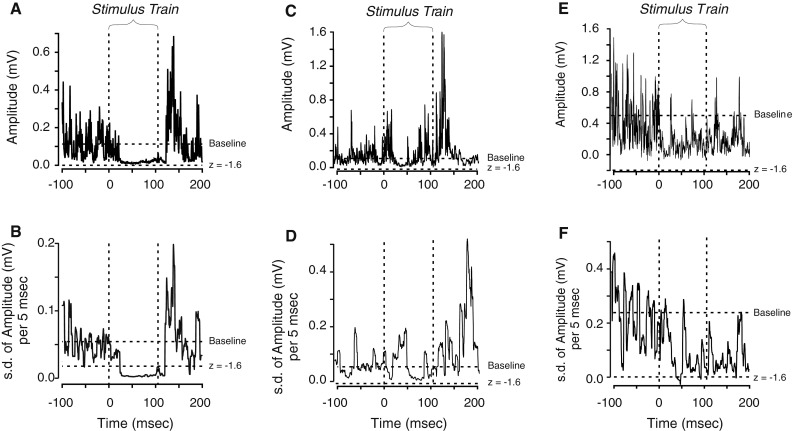
Detecting suppression events. The graphs show the time that the stimulus train started and finished as well as the baseline mean and the *z* score of −1.6. **a**, **c**, and **e** represent the average EMG waveforms; **b**, **d**, and **f** represent the average standard deviation of the EMG responses for a 5 ms moving window. In **a,** the quieter period during the train appears to indicate suppression, but the waveform does not stay below the −1.6 s.d. cutoff line and thus would not be considered a suppression response using a conventional analysis method. In **b,** however, the suppression event is well below the −1.6 s.d. level. Suppression not only reduces the amplitude of EMG, it also reduces its variability. **c** shows a response which was detected by visual inspection; however, the computer analysis (**d**) did not detect the event, so it was not included in the analysis. In **e**, no event was detected on visual inspection, yet computer analysis (**f**) detected the event so it was included in the analysis

To detect suppression, the criteria we chose were a combination of two factors. First, the variability of the EMG level in response to stimulation had to decrease by 1.6 s.d. below the variability observed during baseline (this is comparable to the threshold for a one-tailed *t* test). Second, the actual EMG level during suppression had to be less than the mean EMG level. The specificity for the detection of suppression responses overall was 0.997 (ranging among the subjects from 0.995 to 0.999). The sensitivity for suppression responses was 0.517 (ranging from 0.444 to 0.571). In comparison, the specificity for facilitation responses was 0.993 (ranging from 0.988 to 0.997) and the sensitivity was 0.919 (ranging from 0.891 to 0.948). The sensitivity for suppression could have been improved by making our acceptance criteria more lenient. However, this would have lowered the specificity for suppression, and this was deemed unacceptable. With the criteria chosen, the likelihood of false-positive responses for facilitation and suppression was comparable, and both were less than 1 %. Figure 1 illustrates our approach to suppression detection. Figure 1a illustrates a suppression response that was not detected using the conventional approach but was easily detected by measuring the decreased variability of the EMG level (Fig. 1b) in combination with a slight reduction in EMG amplitude. There were also times when visual inspection was at odds with the computer response detection (Fig. 1c–f). Although the computer detection method did not always agree with visual inspection, these occasions were rare and involved small suppression responses. Thus, the computer detection approach provided an objective measure of events that worked for suppression responses that were readily apparent on inspection, but not detected with the conventional ±2 s.d. approach.

To assess the latency of EMG response onset, a customized script was written using Spike 2 software that allowed us to identify the time when the response left baseline and when it returned to baseline.

### Anatomical reconstruction

After euthanasia and perfusion, the brain tissue was cut coronally on a freezing microtome at 50 μm. Every tenth section was mounted and stained with cresyl violet, for the reconstruction of the stimulation sites. Tissue sections were compared to the Szabo and Cowan atlas (Szabo and Cowan 1984) to locate each motor area. In O, four sites were removed from the analysis because stimulation was in subcortical white matter. In addition, seven sites were removed from O as they crossed the midline during penetrations targeting SMA and had most likely ended up in SMA on the other side.

### Statistical analysis

Statistical analyses were run using Microsoft Excel and SPSS version 19 software packages. Chi-square analysis was used to identify differences between frequencies of responses by stimulation site or by laterality. Kruskal–Wallis analysis was used to compare the onset latencies of EMG responses between all cortical areas, and Mann–Whitney tests were used to compare median threshold currents and EMG onset latency between individual cortical areas. Descriptive statistics were used to further explore differences in response patterns between cortical regions. Criterion level of *p* ≤ 0.05 was used to determine statistical significance, and post hoc Bonferroni corrections were made to account for multiple comparisons within each group.

## Results

### Responses from each area

**Fig. 2:**
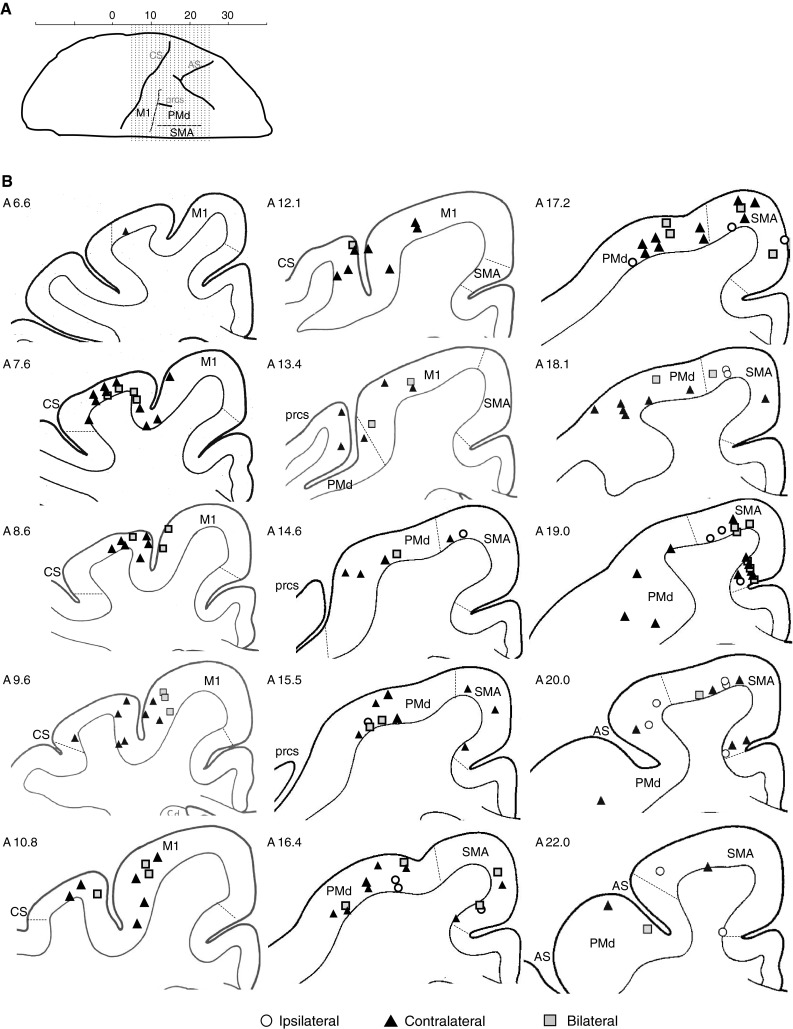
Schematic representation of the cerebral cortex illustrating responsive stimulation sites in each motor area. **a** A cranial view of the *left cerebral cortex* showing the position of M1, SMA, and PMd in relation to physical landmarks (from *left* to *right* is caudal to rostral and the lateral surface is uppermost). **b** The caudal to rostral representation of the location of cortical stimulation points that elicited EMG responses (cortical tracings based on Szabo and Cowan). *White circles* indicate ipsilateral response sites, *black triangles* indicate contralateral sites, and *gray squares* indicate bilateral sites. As shown, most of the ipsilateral sites were located in rostral and medial cortical areas (SMA). *CS* central sulcus, *prcs* precentral dimple, and *AS* arcuate sulcus

**Fig. 3:**
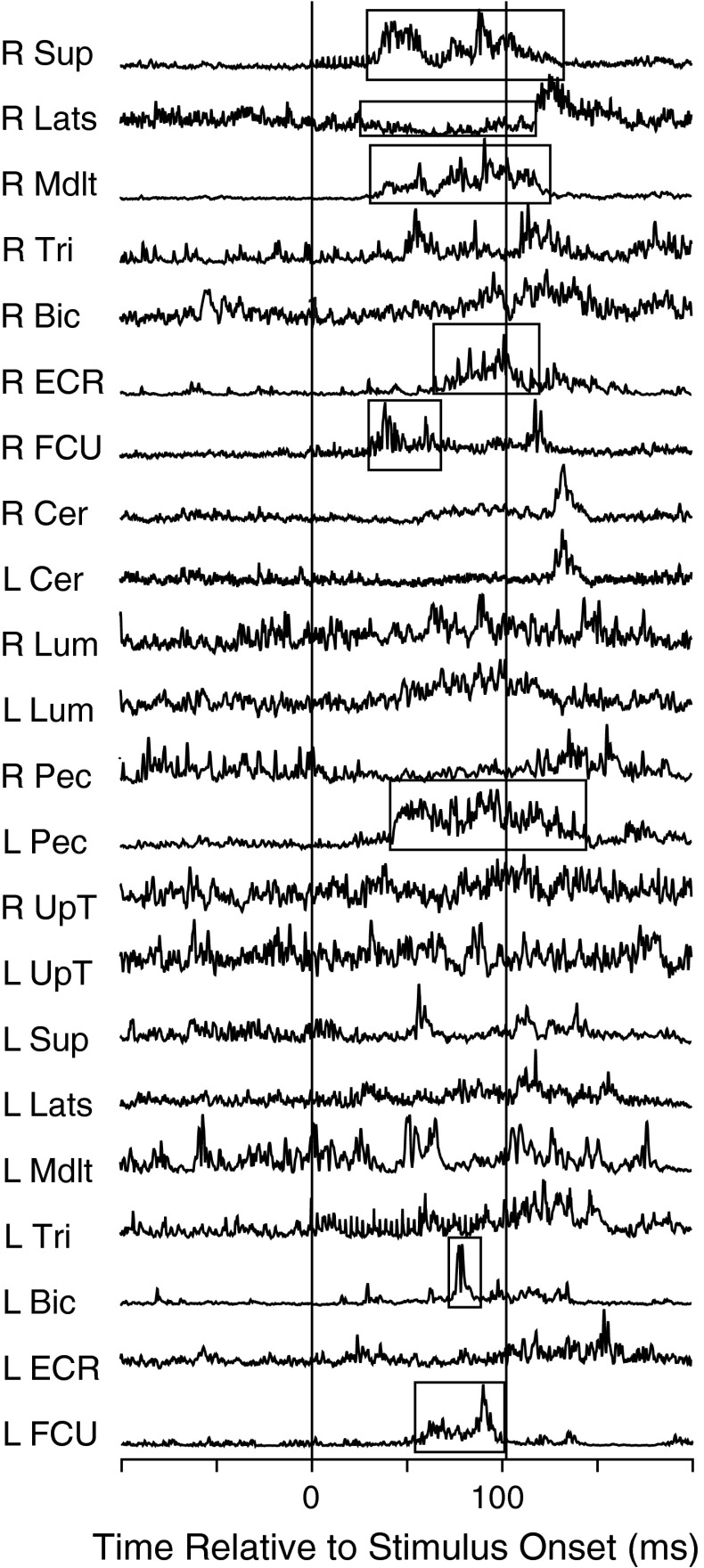
Example of an EMG response from a stimulus train applied in SMA showing EMG responses for 22 muscles. This is a bilateral response with responses seen in both *left* and *right* upper limbs. The *boxes* highlight the objectively detected muscle responses

Among the three subjects, a total of 269 sites were stimulated in the three cortical motor areas (M1, SMA, and PMd), with 109 sites from O, 57 sites from N, and 103 sites from H. Majority of sites were in M1 (110 sites), with 88 sites being in PMd and 71 sites located in SMA. Of these, stimulation of 156 sites produced at least one significant response in EMG. Accepted muscle responses were found from 59 of the 110 M1 sites, 50 of the 88 PMd sites, and 47 of the 71 SMA sites. The location of these responsive sites is illustrated in Fig. 2. Stimulation in each of the three cortical areas resulted in a mixture of ipsilateral, contralateral, and bilateral responses. M1 stimulation evoked the highest number of muscle responses per effective site, averaging 3.9 responses per site, whereas PMd and SMA evoked fewer muscle responses, averaging 2.3 and 2.7 responses per site, respectively. An example of EMG responses from stimulation in SMA is shown in Fig. 3, where muscle responses are highlighted by boxes.

By the final acceptance criteria, 473 significant EMG responses were detected. These came from 58 % (156) of the sites tested. Facilitation was the most common response, with 352 of all 473 muscle responses being facilitation (74 %) and only 121 responses being suppression (26 %). These were distributed evenly throughout the three subjects—O (116 facilitation and 40 suppression responses), N (134 facilitation and 39 suppression responses), and H (102 facilitation and 42 suppression responses).

M1 and SMA had the highest proportion of facilitation events, with 80 % of responses from M1 and SMA being facilitation and 20 % being suppression. In contrast, PMd stimulation caused facilitation in only 58 % of responses and suppression in 42 % of responses. A chi-square analysis showed there were significantly more EMG suppression events following PMd stimulation compared with stimulation of the other two areas (*χ* ^2^ = 21.54, *p* < 0.001). We also compared the proportion of facilitation and suppression events in the ipsilateral and contralateral ULs within each cortical area. In each cortical area, the total proportions were similar, with 80 % facilitation evoked in the ipsilateral UL and 73 % facilitation in the contralateral UL.

As mentioned in the methods, threshold current was used to stimulate each cortical area. As expected, the median current used to stimulate M1 was significantly lower (30 μA) than the median current used in PMd (50 μA, *p* < 0.001) and SMA (70 μA, *p* < 0.001). Within each cortical area, there was no difference between threshold currents required to elicit ipsilateral and contralateral responses.

### Ipsilateral, bilateral, and contralateral responses by cortical motor area

**Fig. 4:**
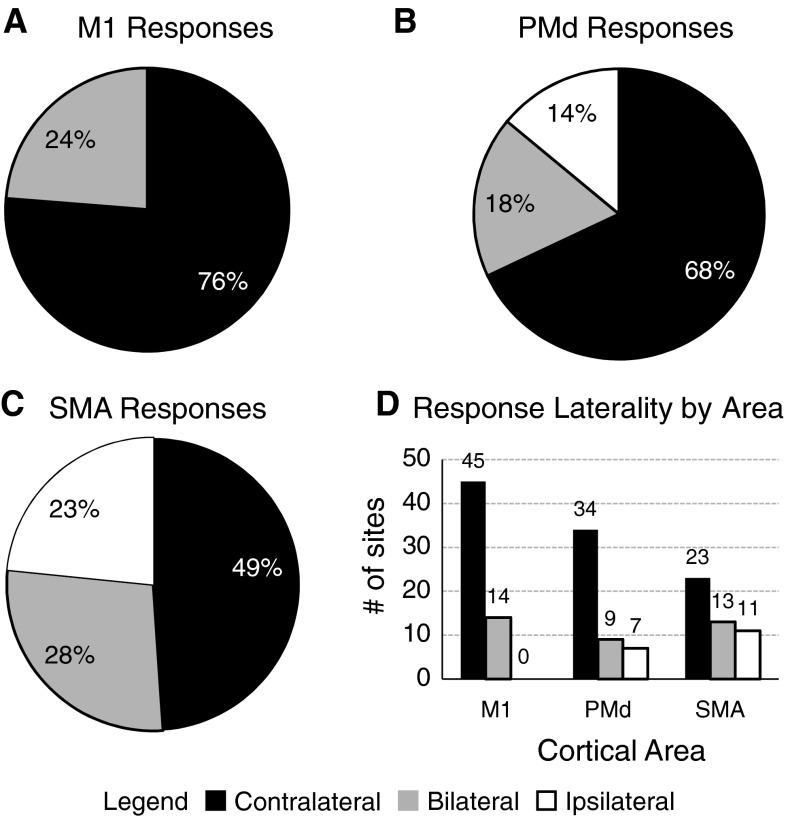
Breakdown of the percentages of muscle responses by laterality for each of the cortical motor areas. M1 stimulation resulted in no ipsilateral responses (**a**); however, stimulation of PMd and SMA (**b** and **c**) resulted in a number of ipsilateral responses (**c**). More than 65 % of sites in M1 and PMd produced contralateral responses (**a** and **b**); however, only 49 % of sites in SMA produced contralateral responses (**c**). The graph in **d** shows the response laterality for each area with the number of sites showing contralateral, ipsilateral, and bilateral responses recorded above each bar. There were more ipsilateral and fewer contralateral sites found in SMA compared with M1 and PMd. For each graph, contralateral responses are represented in *black*, ipsilateral responses are represented in *white*, and bilateral responses are represented in *gray*

Sites where stimulation responses were detected were categorized as producing contralateral (affecting only right UL muscles), ipsilateral (affecting only left UL muscles), or bilateral responses (affecting left and right UL muscles). Only three of the 156 sites (2 %) that showed EMG activity were observed to produce bilateral or ipsilateral movement (one site from M1 and two sites from SMA). As illustrated in Fig. 4, over half of the stimulation sites in M1 and PMd produced only contralateral responses (M1: *n* = 45, 76 %; and PMd: *n* = 34, 68 %). Sites producing ipsilateral responses in these areas were not commonly observed, with no M1 sites and 7 PMd sites (14 %) producing ipsilateral UL muscle activity. In contrast, SMA sites showed a greater propensity for ipsilateral responses, with 11 SMA sites (23 %) producing only ipsilateral activity and 23 SMA sites (49 %) producing only contralateral activity. The proportion of bilateral responses evoked from each of the cortical motor areas was similar, with 24 % of sites in M1 (14/59), 18 % of sites in PMd (9/50), and 28 % of sites in SMA (13/47) evoking EMG responses in both ULs. In all, the proportion of ipsilateral responses resulting from SMA stimulation was higher than the proportion of ipsilateral responses evoked from M1 or PMd stimulation. Conversely, there were significantly fewer contralateral responses evoked from SMA stimulation compared to M1 and PMd stimulation (*χ* ^2^ = 16.87, *p* = 0.002).

Although all three areas had a similar proportion of sites producing bilateral responses, we investigated whether there was a difference in the number of ipsilateral responses detected at bilateral sites. M1 and PMd produced a similar ratio (1:3) of ipsilateral to contralateral responses at bilateral sites. The ratio of ipsilateral to contralateral responses at bilateral sites in SMA appeared to be higher (1:2.1), but this difference was not statistically significant.

### Ipsilateral versus contralateral responses for individual muscles

The comparison above characterized the laterality of responses evoked from individual stimulation sites. In order to estimate the overall role of the three cortical areas, we next examined the total number and proportion of ipsilateral and contralateral EMG responses in individual muscles from each cortical motor area. Of the 231 responses from M1, 196 (85 %) were contralateral and 35 (15 %) were ipsilateral. A similar pattern was observed for PMd; 93 of the 114 responses (82 %) were contralateral and 21 (18 %) were ipsilateral. In SMA, however, a greater proportion of ipsilateral responses was observed; only 84 of the 128 responses (66 %) were contralateral, while 44 (34 %) responses were ipsilateral (*χ* ^2^ = 18.92, *p* < 0.001).

**Fig. 5:**
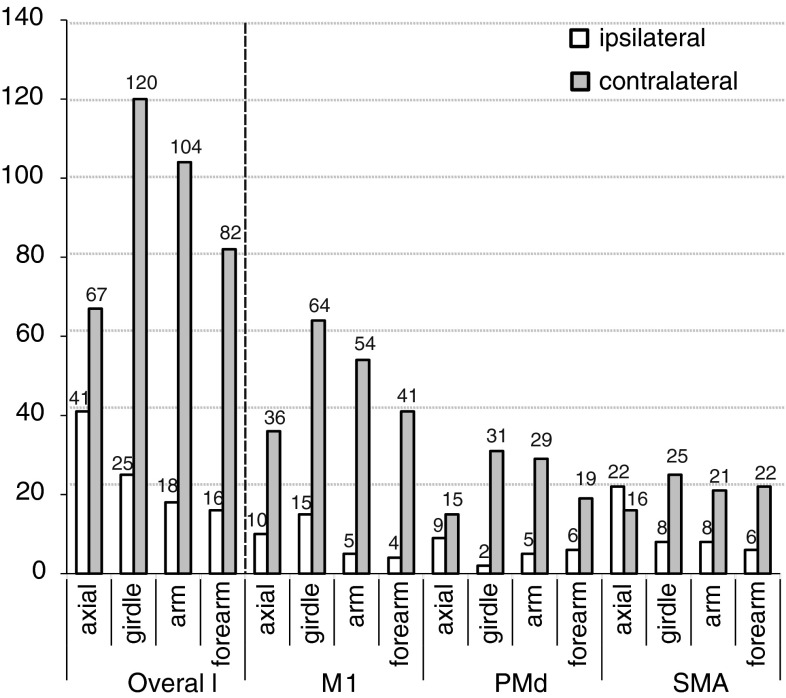
Graph showing the breakdown of ipsilateral and contralateral responses based on UL region for all the cortical areas combined (overall) and for each cortical motor area (M1, PMd, and SMA). The numbers above *each bar* in the graph represent the number of responses detected. The greatest proportion of ipsilateral responses occurred in the most proximal muscle groups (axial region; 38 %) compared with more distal areas, including the girdle muscles (17 %), arm (15 %), and forearm (16 %). The *hatched line* separates the overall responses from those for the individual cortical areas. SMA stimulation resulted in significantly more ipsilateral responses especially in the axial muscle compared to those for M1 and PMd

We also analyzed the data to determine whether the laterality of evoked responses was related to whether muscles were more proximal or distal within the UL. As shown in Table 1, the muscle responses were separated by anatomical regions of the trunk and UL: (1) axial—muscles of the trunk; (2) girdle—muscles around the scapula and shoulder girdle; (3) arm—muscles controlling the shoulder and/or elbow joints; and (4) forearm—muscles in the forearm controlling the wrist. The total muscle responses were evenly distributed between the more proximal (axial and girdle) and more distal (arm and forearm) regions, with 253 of the 473 responses in the axial and girdle regions and 220 responses in the arm and forearm regions. As described previously, of all muscle responses, 373 (79 %) were contralateral and 100 (21 %) were ipsilateral (Fig. 5). Comparing laterality of response by body region revealed that the muscles that were more proximal in the UL (axial and girdle) produced a higher proportion of ipsilateral responses (66/253, 26 %) than those that were more distal (34/220, 15 %). This difference was significant (*χ* ^2^ = 24, *p* < 0.001). Further analysis also revealed there was a difference in the proportion of ipsilateral to contralateral responses in the axial muscles (41/108, 38 %) as compared to the proportion of ipsilateral to contralateral responses in other arm regions (59/365, 16 %). As shown in Table 1, the proportion of ipsilateral responses is higher in the more proximal UL regions for all three cortical areas. This feature is most clearly demonstrated in the responses to M1 stimulation, where only 9 % (4/45) of responses in the forearm was ipsilateral compared to 22 % (10/46) of responses that were ipsilateral in the axial region.

**Table 1:** List of muscles from which EMG was recorded, grouped according to the body region

Arm region	Muscle	All cortical areas		M1		PMd		SMA		Total
		*Ipsi*	*Contra*	*Ipsi*	*Contra*	*Ipsi*	*Contra*	*Ipsi*	*Contra*	
Axial	CervPara	**6**	**6**	4	4	0	2	2	0	12
	LumPara	**13**	**12**	2	5	2	0	9	7	25
	SCM	**4**	**7**	1	6	1	0	2	1	11
	Pec	**18**	**42**	3	21	6	13	9	8	60
	*Proportion*	***38***	***62***	*22*	*78*	*38*	*63*	*58*	*42*	
Girdle	UpTrap	**9**	**24**	4	16	0	6	5	2	33
	Supra	**12**	**36**	7	19	2	8	3	9	48
	Lats	**4**	**60**	4	29	0	17	0	14	64
	*Proportion*	***17***	***83***	*19*	*81*	*6*	*94*	*24*	*76*	
Arm	MDelt	**7**	**31**	2	14	2	8	3	9	38
	Bic	**6**	**24**	3	15	0	6	3	3	30
	Tri	**5**	**49**	0	25	3	15	2	9	54
	*Proportion*	***15***	***85***	*8*	*92*	*15*	*85*	*28*	*72*	
Forearm	FCU	**8**	**41**	2	23	1	6	5	12	49
	ECR	**8**	**41**	2	18	5	13	1	10	49
	*Proportion*	***16***	***84***	*9*	*91*	*24*	*76*	*21*	*79*	
Total		**100**	**373**	34	195	22	94	44	84	473

The numbers in the ipsilateral and contralateral columns are the total number of EMG responses for each muscle over all three subjects. Muscle responses are shown for all cortical areas combined (in bold) as well as for each cortical area independent of the others. The proportion of ipsilateral and contralateral responses is also reported under each muscle region (in italics)

Finally, we tested for differences in the onset latencies of the EMG for contralateral and ipsilateral responses among the motor areas. M1 stimulation produced both the fastest onset times (contralateral responses = 38 ± 20 ms) and the slowest onset times (ipsilateral responses = 58 ± 26 ms). This difference between the contralateral and ipsilateral onset times from M1 stimulation was significant (*H* = 10.26, *p* = 0.001). There was no difference between the onset of ipsilateral and contralateral responses in PMd (contralateral = 42 ± 21 ms and ipsilateral = 47 ± 20 ms) or SMA (contralateral = 52 ± 23 ms and ipsilateral = 44 ± 25 ms). However, there was a significant difference in the onset of contralateral responses from SMA compared to contralateral responses from M1 (*W* = 24876, *p* < 0.001) and PMd (*W* = 7230, *p* = 0.0021). Overall, contralateral responses from M1 and PMd had the shortest latencies, whereas contralateral responses from SMA and all ipsilateral responses had longer latencies. In terms of the onset latency for facilitation and suppression events, suppression responses elicited by M1 stimulation occurred significantly earlier than facilitation responses (facilitation = 43 ms and suppression = 31 ms; *H* = 15.81, *p* < 0.001). This faster suppression from M1 was only significant in the contralateral UL (*H* = 14.93, *p* < 0.001) and was seen in all muscle groups except for muscles of the arm region (axial: *H* = 4.41, *p* = 0.036; girdle: *H* = 5.62, *p* = 0.018; and forearm: *H* = 7.13, *p* = 0.008). As noted above, there was no significant difference in facilitation and suppression response onset latency with PMd and SMA stimulation.

## Discussion

The present study reveals a somewhat higher degree of ipsilateral and bilateral motor outputs from the cortical motor areas than previously described. Most likely, this is a result of three factors. First, and most obvious, we included both the ipsilateral and contralateral limbs in our study. Second, rather than relying completely upon observation, we used EMG measures to reveal changes in muscle recruitment that might not have been visible as movements in response to stimulation. And third, we sampled muscles throughout the limb, especially proximal muscles, rather than focusing on the distal muscles of the forearm and hand. We also found two specific differences in the distribution of motor outputs from the three cortical motor areas. First, outputs from SMA were more likely to be ipsilateral and bilateral than those from M1 or PMd. And second, there was slightly more suppression from PMd than from SMA or M1. Finally, this study is consistent with previous reports in the general finding that from all three cortical motor areas, the predominant response observed was contralateral.

The fact that SMA produces more ipsilateral and bilateral outputs extends results of earlier work showing that SMA is a significant source of ipsilateral and bilateral control of the ULs (Brinkman and Porter 1979; Hoshi and Tanji 2004; Tanji et al. 1988), by demonstrating for the first time that SMA may provide a greater influence over the ipsilateral UL than PMd or M1. Our finding that SMA has a greater influence over the ipsilateral UL when compared to PMd and M1 is important because cortical lesion studies in humans have shown that SMA activity is important in recovery of UL function postinjury (Kimberley et al. 2006; Mintzopoulos et al. 2009). In comparing the three motor areas in the same subject, we also found that PMd stimulation resulted in significantly more suppression events compared with stimulation in the other areas. This finding is interesting in regard to previous findings that have shown that some mirror neurons in the ventral premotor area (PMv) result in suppression of pyramidal neurons (Kraskov et al. 2009). This incidental finding of suppression events elicited from PMd stimulation warrants further study.

Physiological studies of the role of the corticospinal tract in animals have traditionally focused on EMG responses in the contralateral limb (Cheney and Fetz 1985; Fetz and Cheney 1980) and have rarely included comparisons with ipsilateral EMG activity. Often, the only record of ipsilateral muscle activity is through visible observation of ipsilateral movement following stimulation (Kwan et al. 1978; Penfield 1954; Welker et al. 1957). This method has led to few clear observations of ipsilateral movement and thus resulted in the conclusion that ipsilateral corticospinal activity is relatively inconsequential for motor control (Allison et al. 2000; Soteropoulos et al. 2011). Although there have been several studies published with careful mapping of the outputs from M1, PMd, and SMA (Asanuma and Rosen 1972; Boudrias et al. 2010a; Brinkman and Porter 1979; Hummelsheim et al. 1986; Kwan et al. 1978; Rizzolatti et al. 1988), previous studies have not allowed a direct comparison of the effects of identical stimulation trains in all three areas in the same subject. Extrapolating comparisons among studies into a comprehensive picture is difficult. Differences in the degree of neuronal excitability of the M1 and premotor areas are well established, with M1 responding to shorter stimulus trains and lower currents than SMA and PMd (Asanuma and Rosen 1972; Hummelsheim et al. 1986; Mitz and Wise 1987; Weinrich and Wise 1982). The favored neurophysiological approach has been to use the shortest duration stimulus train capable of producing a consistent response (Strick 2002). However, Asanuma and colleagues and Jankowska et al. have shown that repetitive stimulation with longer trains is more effective at producing muscle contraction (Asanuma et al. 1976; Jankowska et al. 1975). Mitz and Wise (1987) found that a 36-pulse stimulus train was required to effectively observe the outputs from SMA. Longer trains have also been used in PMd reports (Hummelsheim et al. 1986; Weinrich and Wise 1982) and some M1 studies (Kwan et al. 1978). In the present study, we used a 36 pulse (105 ms) train in each cortical motor area in order to allow a direct comparison of the evoked effects among all three areas. At each site, the current used was the minimum amount sufficient to consistently produce a visible motor response. The results obtained must be considered with those methodological details in mind.

Another caveat for the reader is that, as noted in the methods, the prevalence of suppression of muscle activity in response to cortical stimulation was most likely underestimated in the present data set. We did use the same criteria among all three cortical motor areas, so this does support the conclusion that suppression was more prevalent from PMd than from M1 or SMA. However, our criteria were aimed at specificity, not sensitivity. Comparisons of the overall prevalence of suppression from this study should be interpreted with that in mind.

We note here that, by observations made during the studies, contralateral UL movement was often observed and ipsilateral UL movement was rarely seen. Because we relied upon EMG analysis rather than simple observation, we demonstrate here for the first time that ipsilateral muscles are being recruited even at times when ipsilateral UL movement was not observed. Notably, many of the ipsilateral muscle responses occurred in conjunction with contralateral muscle responses, creating a bilateral pattern that has not been documented previously. In addition, we have demonstrated that the majority of recorded ipsilateral responses occurred in proximal musculature around the trunk and shoulder girdle. Movement in this area is harder to observe than that occurring more distally and may have been overlooked in previous studies that relied on the observation of muscle activity.

Our findings that 21 % of responses to stimulation are ipsilateral seem to contradict earlier physiological studies (Soteropoulos et al. 2011; Tanji et al. 1988; Tanji and Kurata 1981), which found < 4 % of responses was ipsilateral. Our proportion of ipsilateral responses is also higher than would be predicted from tract tracing studies. Only 10–13 % of all corticospinal fibers descends ipsilaterally from the motor areas directly to the cervical spinal cord (Lacroix et al. 2004; Rosenzweig et al. 2009; Yoshino-Saito et al. 2010), although the proportion of ipsilateral terminations from SMA is closer to 23 % (Dum and Strick 1996). We theorize that this is due to our use of longer trains that activate both monosynaptic and polysynaptic pathways (Asanuma et al. 1976; Jankowska et al. 1975; Patton 1982) and our focus on more proximal muscles of the trunk and UL. Earlier studies have shown that ipsilateral and bilateral activities are more common in proximal muscles than in distal muscles (Bawa et al. 2004). Thus, we would expect a study with a strong representation of proximal muscles to have good success revealing ipsilateral influences. The control of the trunk and shoulder is imperative in order to allow wrist and hand movements to occur, so including these muscles is important for understanding the role that the ipsilateral cortex may have on the control of UL movement. And as noted, stimulus trains can effectively activate both monosynaptic and polysynaptic pathways (Asanuma et al. 1976; Jankowska et al. 1975; Patton 1982). Thus, a number of these ipsilateral responses may have resulted from interhemispheric transcallosal pathways, corticoreticulospinal pathways through brainstem motor nuclei, or pathways at the segmental level via commissural spinal interneurons. Interestingly, studies have shown that although M1 and PMd have transcallosal projections to homologous regions of the opposite cortex, SMA projects to multiple motor areas in the opposite cortex, including SMA, PMd, and M1 (Fang et al. 2008; Liu et al. 2002). Such transcallosal projections may explain our findings that SMA produces more ipsilateral and bilateral responses than M1 and PMd, because of the wider effects that SMA has on the contralateral motor cortex. Studies have also shown that the premotor areas also send collateral projections to brainstem nuclei such as the pontomedullary reticular formation (Keizer and Kuypers 1984, 1989; Matsuyama and Drew 1997; Rho et al. 1997), and it is well known that commissural interneurons in the spinal cord are often involved in efferent circuits that influence muscle activity (Jankowska and Edgley 2006; Jankowska and Stecina 2007). The fact that higher currents were required to elicit responses from PMd and SMA, compared with the currents required for M1, implies that these premotor areas are more likely to use such indirect pathways involving interhemispheric, brainstem, and/or spinal circuits (Jankowska 1975).

Although there was no difference in the threshold currents required to elicit ipsilateral or contralateral responses, the onset of ipsilateral responses was significantly later than those of contralateral responses. This delay in response for ipsilateral muscles indicates that the ipsilateral responses were elicited either through direct pathways involving small, lightly myelinated fibers or through indirect polysynaptic pathways such as those discussed above. We also found that the latency for suppression was somewhat faster than that for facilitation with M1 stimulation. We suspect that this is due to the general neurophysiological principle that inhibitory synapses tend to be somatic, so inhibition may require less temporal summation and become evident sooner within the train of stimulation. Why this was true for M1 but not PMd or SMA is unclear, but could be an effect of more direct pathways for M1. The design of this study cannot address which of these mechanisms is responsible for our findings. Our hypothesis is that the present results reflect an amalgamation of all of these motor pathways with both direct and indirect pathways being recruited by the stimulus train. It would be interesting to employ retrograde transsynaptic tracer injections to muscles which produced the most ipsilateral responses as a strategy to identify these polysynaptic pathways. It would also be interesting to repeat this study comparing responses to short versus long stimulus trains to see whether the prevalence of ipsilateral and bilateral responses was markedly affected.

The current study provides definitive electrophysiological evidence that primary and premotor cortical areas can both facilitate and suppress muscle activity in the ipsilateral and contralateral UL and identifies clear differences between influences of the three cortical regions. Stimulation of all three regions generates activity in both ULs, but SMA provides the greatest ipsilateral input to the activity of the muscle groups examined, and PMd activity appears to provide more suppression of EMG than M1 or SMA. These findings demonstrate a functional substrate for ipsilateral cortical control of UL movements in the primate. Further studies of the interactions of these systems may provide a basis to develop strategies for improved motor control of UL movements following injury or damage to the cortex.
